# Biophysical separation of *Staphylococcus epidermidis* strains based on antibiotic resistance

**DOI:** 10.1039/c5an00906e

**Published:** 2015-06-18

**Authors:** Paul V. Jones, Shannon Huey, Paige Davis, Ryan McLemore, Alex McLaren, Mark A. Hayes

**Affiliations:** a Arizona State University , Department of Chemistry and Biochemistry , Tempe , AZ 85287 , USA . Email: mhayes@asu.edu ; Fax: +(480) 965-2747 ; Tel: +(480) 965-2566

## Abstract

Electrophoretic and dielectrophoretic approaches to separations can provide unique capabilities. In the past, capillary and microchip-based approaches to electrophoresis have demonstrated extremely high-resolution separations. More recently, dielectrophoretic systems have shown excellent results for the separation of bioparticles. Here we demonstrate resolution of a difficult pair of targets: gentamicin resistant and susceptible strains of *Staphylococcus epidermidis*. This separation has significant potential implications for healthcare. This establishes a foundation for biophysical separations as a direct diagnostic tool, potentially improving nearly every figure of merit for diagnostics and antibiotic stewardship. The separations are performed on a modified gradient insulator-based dielectrophoresis (g-iDEP) system and demonstrate that the presence of antibiotic resistance enzymes (or secondary effects) produces a sufficient degree of electrophysical difference to allow separation. The differentiating factor is the ratio of electrophoretic to dielectrophoretic mobilities. This factor is 4.6 ± 0.6 × 10^9^ V m^–2^ for the resistant strain, *versus* 9.2 ± 0.4 × 10^9^ V m^–2^ for the susceptible strain. Using g-iDEP separation, this difference produces clear and easily discerned differentiation of the two strains.

## Abstract 2

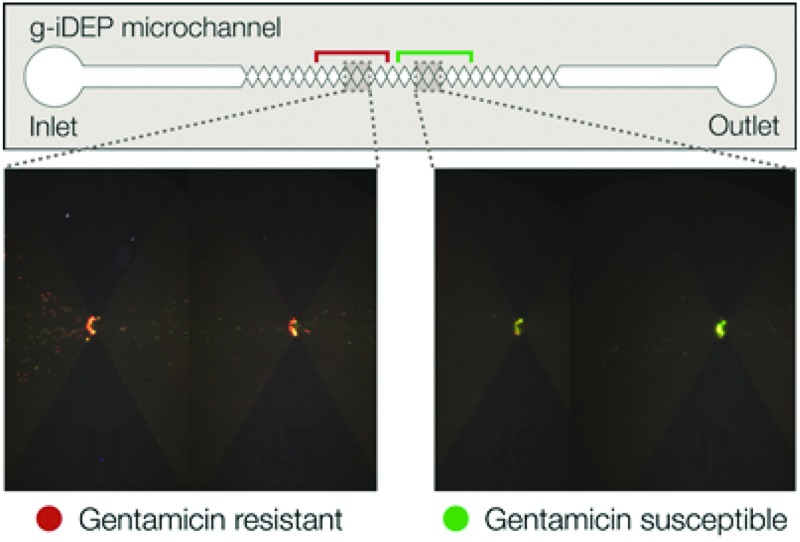
Gradient insulator-based dielectrophoresis used to generate separation and concentration of *Staphylococcus epidermidis*, gentamicin-resistant and susceptible strains.

## Introduction

### Antibiotic resistance in bacteria

Bacteria have developed complex relationships with humans: interactions that span the range of commensalism, mutualism, and antagonism. They have evolved to rapidly develop and exchange beneficial genomic alterations.^1^ One type of adaptation is resistance to antibiotics. Even before the widespread usage of penicillin in the late 1940's, researchers noted that certain bacteria seemed to destroy the drug through enzymatic action.^2^ Resistant strains result in prolonged illnesses and higher mortality rates.^3^ National summary data from the Centers for Disease Control and Prevention (CDC) indicate that each year in the United States, at least two million people acquire serious infections with antibiotic-resistant strains of bacteria. At least 23 000 people die as a direct result of these infections and many more die from related complications.^4^

The genus *Staphylococcus* is represented by some of the most notorious antibiotic resistant strains and species.^5^ These bacteria are spherical, gram-positive, non-motile, facultative anaerobes. They are typically classified as pathogenic or non-pathogenic based on production of the enzyme coagulase. *Staphylococcus epidermidis* does not produce coagulase, and it is generally less invasive than *S. aureus*. In fact, it is a normal and commensal resident of human skin and mucosa.^6,7^ In recent decades, *S. epidermidis* has increasingly emerged as a cause of multi-resistant nosocomial infections.^8^ Immunocompromised patients, indwelling medical devices, and surgically implanted prostheses provide suitable environments for *S. epidermidis* to propagate and form biofilms.^9^ In recent years, it has become the most common cause of medical device-associated colonization and infection.^10^

Strains of *S. epidermidis* have developed resistance to many antibiotics. This research focuses on gentamicin resistance in *S. epidermidis*. Gentamicin is a common aminoglycoside antibiotic. Its mechanism of action (common to all aminoglycosides) results from binding to the 16S subunit of the bacteria's ribosomal RNA (rRNA) and disrupting the protein-proofreading function.^11^ Accumulation of mistranslated proteins interferes with proper cellular function and eventually leads to cell death. Aminoglycoside resistance in gram-positive bacteria occurs through modification of antibiotic *via* aminoglycoside-modifying enzymes.^12^ While the specific case of *Staphylococcus epidermidis* resistance to gentamicin has not been well characterized, gentamicin resistance in the genus *Staphylococcus* in general has been attributed to three specific enzymes: an acetyltransferase, a phosphotransferase, and an adenyltransferase. The enzymes may be present individually or together. Most often, the production of a bifunctional enzyme AAC(6′)-APH(2′′) from the gene *aac*(6′)-*aph*(2′′) is responsible.^13^ The possible mechanisms of resistance in these bacteria must then result from phenotypic changes due to the expression of these enzymes.

Bacteria readily share beneficial DNA through horizontal gene transfer.^14^ Many resistance genes are encoded in plasmid DNA. Transfer of resistance to multiple compounds has been shown to occur through plasmid exchange in natural environments, even between phylogenetically diverse populations.^15^ It is therefore reasonable to assume that the resistance mechanisms found in other bacteria, and especially in other *Staphylococci*, are found in *S. epidermidis* as well.

**Fig. 1:**
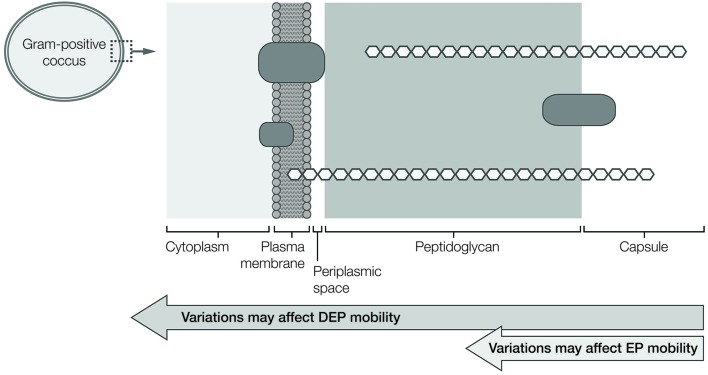
Basic illustration of a gram-positive bacterium. Certain simplified physical components of the bacterium are listed. Changes in any of these components could alter the effective electrostatic and dielectric properties of the cell. The possible effect of these changes on EK and DEP forces are categorized and listed.

The electrostatic and dielectric properties of the bacteria may be influenced by the molecular mechanisms of antibiotic resistance. Biological material in all shapes and sizes is composed of electrostatically interacting atoms, molecules, polymers, and other higher-order structures. Even net-neutral biological particles will possess a unique distribution of charge. The electrostatic, dipolar, and multipolar diversity of all matter presents a valuable mode of manipulation and separation, which is exploited here for the separation of antibiotic-resistant and susceptible bacteria (Fig. 1).

### Electrokinetic forces used for separations

Various methods have been used for cell separation.^16–18^ Dielectrophoresis has emerged as a powerful tool for bioparticle separations. It is important to note that separations using these forces grow from early (and recent) cell characterizations using dielectrophoresis and impedance.^19–33^ A prime example of separations is DEP-based field flow fractionation of cancer cells.^34^ Other examples include separating cancer cells, stem cells, various bacterial cells, infected and healthy red blood cells, platelets and whole blood, and fetal cells from maternal blood.^35–39^ Dielectrophoresis can also be used to separate viable from nonviable cells, as has been shown with both yeast and bacteria.^40^ In this last case, the difference in membrane conductivity was assigned as the reason for separation.

**Fig. 2:**
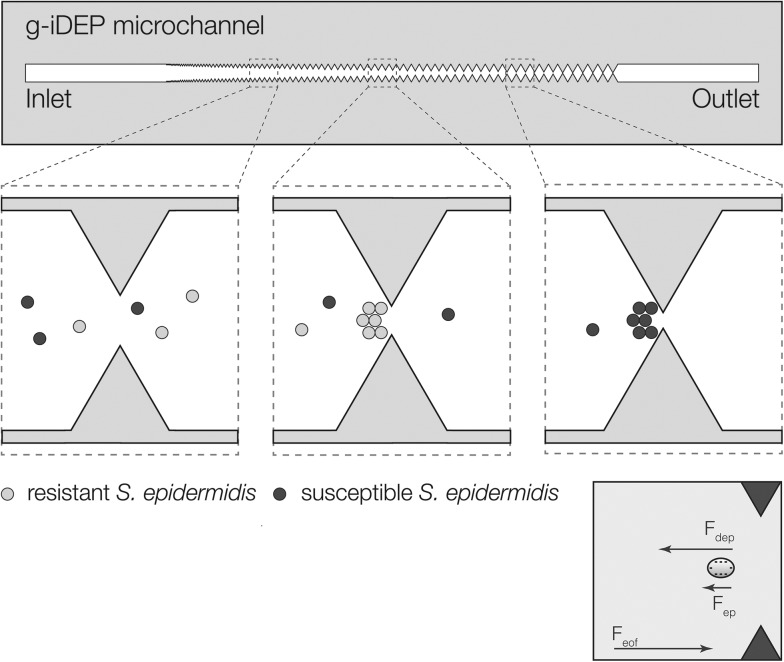
(Top) Conceptual illustration of g-iDEP device and expected capture behavior for *S. epidermidis* resulting from a superposition of opposing forces. The g-iDEP microchannel is patterned in insulating materials and constructed using soft lithography. The geometry consists of a sawtooth pattern: constrictions of gradually decreasing pitch formed by approaching apices of equilateral triangular units. (Middle) Different analytes are expected to capture at unique gates based upon their characteristic EK and DEP properties. In this case, both analytes pass the initial, large-pitched gates unhindered since EK force exceeds DEP force for both types. When gates become sufficiently small-pitched, EK force is overcome by DEP force for one of the two analytes, causing selective capture and concentration. The remaining analyte will continue to progress down-channel. Eventually EK force is overcome by DEP force for the second analyte, resulting in its capture. (Bottom) This shows a basic illustration of relative EK and DEP forces expected to act upon a bacterium traveling along the channel centerline.

The current work is focused on applying gradient insulator-based dielectrophoresis (g-iDEP) to high-resolution separation of pathogens. The mechanism and forces involved in this approach are well described elsewhere.^41–45^ Briefly, g-iDEP systems utilize a continuous microchannel patterned with sequentially changing, constrictive insulating features. These constrictions, referred to as gates, create a series of DEP-inducing electric field non-uniformities. The specific geometry of the channel yields increasingly strong DEP forces along the channel. Particles traveling through the microchannel are propelled by a combination of EP and EOF forces. Since DEP forces scale differently with the channel's geometry than do EP and EOF forces, unique traps are formed at each gate as they become sequentially narrower. This causes physically distinct analytes to settle into discrete zones or balance points near different gates. These collected species do not block the channel; they are held in place by the induced forces. Other particles can freely pass through the collection zones. They assume unique positions along the channel's separatory axis based on their electrophoretic (*μ*_EP_) and dielectrophoretic (*μ*_DEP_) mobilities (Fig. 2). Considered together, a particle's electrophoretic and dielectrophoretic mobilities reflect an array of properties including size, charge, polarizability, shape, and heterogeneity.^46^ Interrogating all these properties together yields a separatory scheme that is fine-tuned for high-resolution capture and concentration of pathogens. In assessing the work here, the most important relationship is 
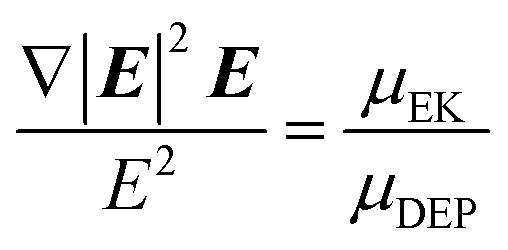
, which occurs at the balance or focusing point for the particles and ***E*** is the electric field vector.

### Significance of g-iDEP separations

Current clinical approaches to determination of antibiotic resistance often require two or more days to obtain results. They typically rely upon treating the bacteria with antibiotics, then observing colony growth patterns.^47^ The long turnaround times lead to increased reliance upon broad spectrum antimicrobials and generally lead to suboptimal outcomes for patients (including increased mortality rates).^48–50^

The work described here will aid in the creation of rapid diagnostic devices that exploit this high-resolution isolation and concentration of specific and unique bacterial strains. Rapid and early detection will significantly improve therapeutic outcomes. Furthermore, treatment can be based upon more accurate and complete information, facilitating a specific and appropriate response. The physicality and cost of the strategy described here are conducive to the development of devices that could be used in low-power surveillance modes or that could be distributed in low-resource settings. Such applications could impact the spread of disease and tracking of outbreaks.

We report rapid and reproducible differentiation of gentamicin-resistant and gentamicin-susceptible strains of *S. epidermidis*. With appropriate channel design, we demonstrate that simultaneous spatial separation and concentration of these bacterial strains is achievable. This work represents significant progress in demonstrating the ability of g-iDEP to separate nearly identical pathogens.

## Materials and methods

### Microdevice fabrication

Two versions of a sawtooth microchannel were used in these experiments: one for single-strain experiments (V1), and another for dual-strain separations (V2). The former has been described in detail in prior publications.^41–45^ Both versions share core characteristics. In both cases the channel geometry is bounded by adjoined equilateral triangular units; a series of these shapes defines two of the channel walls. Gates are formed where the aligned tips of these opposing triangles approach one another. The triangular units increase in size from inlet to outlet, causing gate pitch to gradually decrease along the length of the channel. Circular, terminal reservoirs serve as the inlet and outlet of the microchannel.

For V1 microchannels, the channel length, width, and depth were 4.1 cm, 1000 μm, and 14 ± 1 μm (average between templates), respectively. The initial gate pitch was 945 μm and the final gate pitch was 27 μm. For V2 microchannels, the channel length, width, and depth were 4.2 cm, 1000 μm, and 20 μm, respectively. The initial gate pitch was 73 μm, and the final gate pitch was 25 μm.

The microfluidic devices described above were fabricated using one of two common soft-lithography strategies.^51^ Channels were patterned on 4 inch Si wafers with AZ P4620 photoresist (AZ Electronic Materials, Branchburg, NJ) and contrast enhancement material CEM388SS (Shin-Etsu MicroSi, Inc., Phoenix, AZ). A high-fidelity chrome photomask was used to expose the photoresist, and then it was developed. Alternatively, wafers were coated with AZ 4330 photoresist (AZ Electronic Materials, Branchburg, NJ). Photoresist was exposed using a glass chrome mask produced by JD Photo-Tools (United Kingdom). After developing, wafers were etched using reactive ion etching (ICP etcher, SPTS, San Jose, CA), with SF_6_ gas and C_4_F_8_ gas.

After preparing the template wafers, polydimethylsiloxane (PDMS) (Sylgard 184, Dow/Corning, Midland, MI) was poured across the wafers, and then cured at 70 °C for one hour. Resulting PDMS casts were then peeled from the templates, trimmed, and punched with 2 mm diameter access holes through the terminal channel reservoirs.

Devices were assembled by bonding PDMS casts to a glass coverplate. Both materials were treated with oxygen plasma in a plasma cleaner (PDC-32G, Harrick Plasma, Ithaca, NY). Treatment with oxygen plasma lasted for 60 seconds at 18 W. The PDMS and glass were then allowed to seal upon contact. This created microfluidic channels with three walls of PDMS and one of glass.

### Cell culture and labeling

Two strains of *Staphylococcus epidermidis* were obtained, including gentamicin resistant (ATCC 35983) and gentamicin sensitive (ATCC 14990) strains. *S. epidermidis* seed stock was stored in tryptic soy broth (TSB) with 10% glycerol at –80 °C. Aliquots of 8 mL sterile TSB were placed in culture tubes. Each tube was inoculated with one of the strains then placed in a shaker/incubator and allowed to grow overnight at 37 °C. Cultures reached late log phase, with a cell concentration of approximately 10^9^ CFU mL^–1^. Following incubation, a 1 : 10 dilution of each cell culture was centrifuged at 4000*g* for 3 minutes. After discarding the supernatant, the cell pellet was resuspended in 1 mL of 2 mM phosphate buffer (PB), pH 7.4, by agitation with a vortexer ensuring redispersion of cells. This process was repeated three times in order to remove all of the TSB.

For single-strain experiments, cells were labeled using Vybrant DiO fluorescent dye (Invitrogen). Excitation and emission wavelengths for this dye are 484 and 501 nm. A 5 μL aliquot of dye was added to each 1 mL suspension of washed cells. Following addition of dye, the sample was mixed with agitation and then incubated in a 37 °C water bath for approximately 20 minutes. Samples were then centrifuged and washed three more times to eliminate unbound dye. Cells were resuspended in phosphate buffer containing 4 mg mL^–1^ bovine serum albumin (BSA). Throughout the process, precautions were taken to minimize exposure to ambient light and thus minimizing photobleaching. After labeling was complete, cells were examined using bright field and fluorescence microscopy to ensure that they were both dispersed and intact.

For dual-strain separations, each strain of *S. epidermidis* was separately labeled with either NHS-rhodamine or NHS-fluorescein (respective excitation/emission wavelengths: 552/575 nm and 494/518 nm). In each case, 1 mg of dye was first dissolved in 100 μL dimethylsulfoxide. A 20 μL aliquot of this mixture was added to 1 mL of washed and suspended bacterial cells. This suspension was incubated in a 37 °C water bath for 20 minutes before washing the cells as described above, and finally suspending them in 1 mL PB with BSA.

### Experimental

A completed microdevice was placed on the stage of an Olympus IX70 inverted microscope with ×4 and ×10 objectives. Labeled bacteria were introduced into the microdevice by pipetting ∼20 μL of cell suspension into the inlet reservoir. Hydrodynamic flow was balanced by pipetting a similar volume of buffer into the outlet reservoir and observing particle motion within the channel. Platinum electrodes with a diameter of 0.404 mm (Alfa Aesar, Ward Hill, MA) were inserted through the PDMS access ports into the terminal reservoirs. They were then connected to a HVS448 3000D high voltage sequencer (Labsmith, Inc., Livermore, CA). A mercury short arc lamp (H30 102 w/2, OSRAM) was used to illuminate the samples. An Olympus DAPI, FITC, Texas Red triple band-pass cube (Olympus, Center Valley, PA) was used for fluorescence microscopy.

In all experiments, bacteria were captured in PB with BSA. The conductivities of these solutions were approximately 343 μS cm^–1^. For single-strain experiments, DC potentials applied across the device ranged from 0–3000 V in 100 V increments. For dual-strain experiments, DC potentials ranged from 800–1200 V in 100 V increments.

For the single-strain experiments, still images and video were collected with a monochrome QICAM cooled CCD camera (QImaging, Inc., Surrey, BC) and Streampix V image capture software (Norpix, Inc., Montreal, QC). For the dual-strain separations, color video data was captured using an iPhone 5S camera. Software included Apple iPhoto for retrieving data from the device, ImageJ for file conversion and fluorescence intensity analysis, and Adobe Photoshop for assembly of channel-wide photo mosaics.

The data were obtained over a period of several months. PDMS casts were kept in airtight plastic bags in the freezer for up to two weeks before use. Casts were bonded to their glass coverplate on the same day they were used for experiments, and were discarded after use. Bacterial preparations were typically labeled and used the day after inoculation and incubation. Prior to fluorescence intensity analysis, the collected imaging datasets were examined to find those showing the least degree of bacterial aggregation and device fouling. For each strain, at least four datasets were used, with each dataset representing separate device and bacterial preparation.

### Mathematical modeling of device

Finite element, multiphysics software (COMSOL, Inc., Burlington, MA) was used to model the electric field within the microchannel. An accurately-scaled 2D geometric representation of the main channel was imported from AUTOCAD. Using a 2D approximation greatly simplifies the calculations and reduces computation time. Although the surface charge of the glass and PDMS surfaces likely differ by some amount, the electrical potential is assumed to vary minimally across the relatively small depth of the microchannel.

### Safety considerations

Organisms used in this experiment were Biosafety Level (BSL) I or II. All experiments were carried out in an approved BSL II laboratory within accordance to the current version of the CDC/NIH BMBL publication.

## Results

The electrokinetic and dielectrophoretic behavior of two strains of *S. epidermidis* were investigated with g-iDEP. Two sets of studies were performed. The first set involved single strains in separate V1 devices. When varying the applied voltage, in V1 microchannels, capture only occurred at the ultimate or penultimate sets of gates. This design was well-suited for single-gate, single-analyte experiments. The second set of studies involved two strains within a single channel, using the V2 design microchannels. The strains were observed simultaneously within the same microchannel with differential labeling. The V2 microchannels feature more incremental stair-steps between sets of gates. The gradual decreases in gate size produce smaller increases in local force maxima and increase the resolving capabilities of the channel.^52^ For this reason, V2 microchannels were used for simultaneous dual-analyte separation.

### Single-strain experiments

**Fig. 3:**
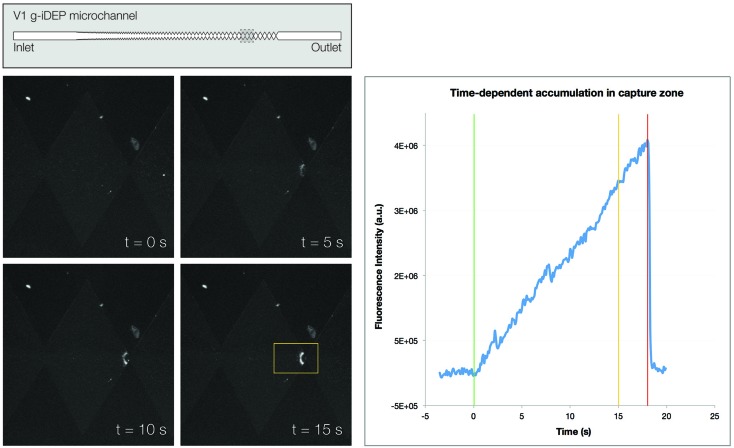
Capture of gentamicin-resistant *S. epidermidis* at a 27 μm gate within a V1 microchannel. Material is captured and concentrated in tight, crescent-shaped bands near the gate. Above the threshold value of *V*_A_ required for capture, bacteria collect continuously as long as potential is applied. (Left) Images show capture at four different time points when *V*_A_ = 1200 V. ROI is framed in yellow for the bottom left image. (Right) Integrated fluorescence intensity over the ROI shows steady accumulation of bacteria. The green line indicates *t*_A_ = 0 s, or the point when potential was applied. The yellow line indicates *t*_A_ = 15 s, or the point at which FI was measured for subsequent analysis of *V*_A_-dependence of capture. The red line indicates the point at which potential was removed.

The magnitude (*V*_A_) and duration (*t*_A_) of applied electric potential were varied. The overall behavior of the bacteria was consistent with the results of previous work, using gates of similar geometry. Upon application of an electric potential within the device, motion of all analyte was directed towards the outlet reservoir (cathode), consistent with EOF-dominated transport. No capture was observed at gates with pitch greater than 90 μm. Analyte behavior was examined at the final set of gates (27 μm pitch). At the gate of interest, capture resulted in the formation of crescent-shaped bands material, localized immediately upstream (within a few micrometers) of the gate's transverse axis of symmetry (Fig. 3, left).

The amount of material that accumulated within the capture zone depended upon both the magnitude and duration of applied potential. Accumulation was quantified by integrating fluorescence intensity (FI) across a small region of interest (ROI) centered at the point of typical band formation. Below strain-specific threshold values (*c*) of *V*_A_, no capture occurred, even over extended periods of time. Above this threshold value of *V*_A_, material continued to accumulate as long as potential was maintained. Under these conditions, FI within the ROI increased linearly with *t*_A_ (Fig. 3, right).

**Fig. 4:**
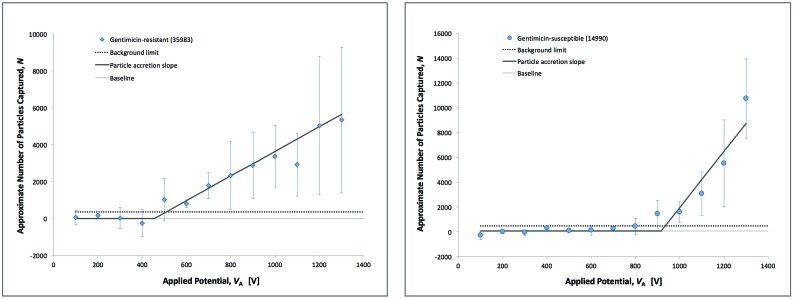
Plots of captured particle count for both gentamicin-resistant (left) and gentamicin-susceptible (right) *S. epidermidis*, with increasing applied potentials (*V*_A_). All data was collected at a 27 μm gate on V1 microchannels, with a duration of applied potential (*t*_A_) of 15 seconds. Accumulation was noted when particle count exceeded the background limit (twice the standard deviation of baseline data points). Lines are fitted from the data. Intersection of the sloped line and the baseline give a more precise estimate of the threshold at which capture is initiated (see text).

Data was examined at a consistent time point (*t*_A_ = 15 s, yellow line in Fig. 3, right) across a range of voltages from 100 to 2000 V in 100 V increments, for both strains of *S. epidermidis* (Fig. 4). Integrated FI values for the ROI were then divided by the mean FI signal for individual, labeled bacteria in order to convert these values to approximate particle count (*N*).

In order to estimate the threshold (*V*_A_ = *c*) at which capture occurs, the characteristics of baseline behavior were first determined at low values of *V*_A_. Specifically, the baseline for each strain was established by averaging the results measured from 100–400 V. Calculating the baseline magnitude and variation in this manner then allowed determination of statistically significant signal resulting from capture. This was noted as the first value of *V*_A_ for which the magnitude of *N* exceeded two times the standard deviation of the average baseline value.

Signal was generated when the applied voltage was sufficient to generate trapping force. As *V*_A_ increased (*V*_A_ *> c*), the amount of material accumulated during the 15 s window increased. This yielded a predominantly linear, positive slope for particle count at values of *V*_A_ greater than *c*. Since the transport and capture mechanisms are known, the increased intensity at higher values of *V*_A_ can allow for more accurate estimation of *c*. This behavior is well described as a piecewise function, where the *y*-axis represents *N*, and the *x*-axis represents *V*_A_. The general form of this relationship is as follows:1
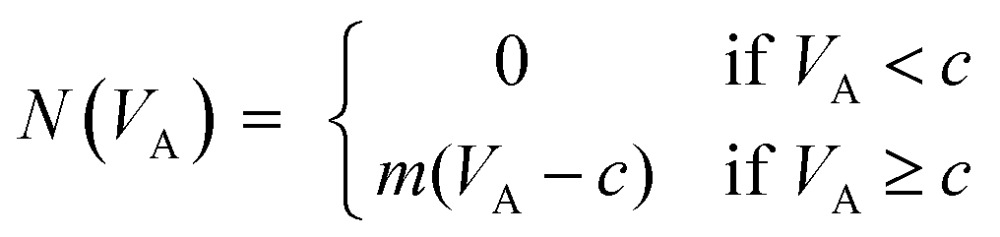



Assuming that a large proportion of the particle population is successfully trapped within a capture zone, the slope of this line (*m*) is primarily related to the rate of analyte delivery to the gate. Once established or estimated, the specific value of *c* is related to the values of *μ*_EK_ and *μ*_DEP_ intrinsic to an analyte population, and can be described in relation to the electric field parameters and in terms of the ratio of the two mobilities (*μ*_EK_/*μ*_DEP_).

Data points above the estimated value of *c* were fitted using linear regression. The slope and intercept of these lines were used to determine the rate of particle accumulation and extrapolate values for *c* where the accumulation slope intersected the baseline. In this manner, values for *c* were determined to be 443 ± 59 V and 881 ± 38 V along the *x*-axis for the resistant and susceptible strains, respectively. Using COMSOL models, the equivalent ratio was determined to be 4.6 ± 0.6 × 10^9^ V m^–2^ for the resistant strain *versus* 9.2 ± 0.4 × 10^9^ V m^–2^ for the susceptible strain.

### Dual-strain experiments

**Fig. 5:**
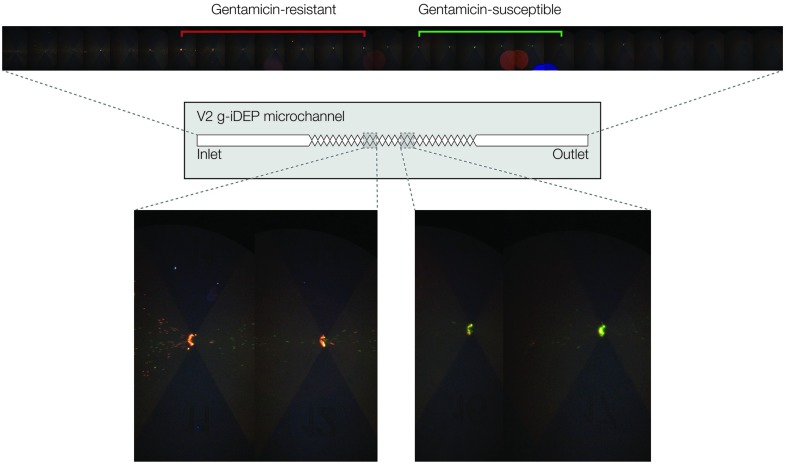
Images showing simultaneous capture and concentration of gentamicin-resistant (red) and gentamicin-susceptible (green) *S. epidermidis* within separate regions of a single microchannel. (Top) An image mosaic of the V2 microchannel shows that capture is distributed across several gates for each strain. Approximately 8 gates separate the mean gate position for each strain's region of capture, with mixing at some of the intervening gates. (Bottom) Detailed images taken from different regions of the channel show the formation of selective capture zones for each strain.

A significantly different channel design (referred to as V2) was used for a simultaneous study of gentamicin-resistant and susceptible *S. epidermidis*. Values of *V*_A_ ranged between 800 and 1200 V in 100 V increments. In each experiment, significant differences in behavior were noted for the resistant (red-labeled) and susceptible (green-labeled) bacteria (Fig. 5). There was a distinct capture of red particles at larger gate pitch and green particles at smaller pitch. There was considerable spread in the loci of collection and notable overlap where both red and green were observed at some gates. These general observations held for all *V*_A_ where capture was observed, with capture occurring at smaller gates with lower *V*_A_. The largest differentiation between strains was observed at *V*_A_ = 1000 V. The observed capture behaviors were consistent with the findings from single-strain experiments. Namely, the strain exhibiting lower mobility ratio (gentamicin-resistant) was captured at larger-pitch gates relative to the strain exhibiting higher mobility ratio, which was captured at smaller-pitch gates for any given value of *V*_A_.

## Discussion

A new micro-scale separation technique was used to generate high-resolution isolation and concentration of gentamicin-resistant and gentamicin-susceptible strains of *Staphylococcus epidermidis*. By most metrics these two strains are phenotypically identical, thus presenting a significant challenge to traditional analytical separation techniques. Using g-iDEP microchannels, the strains were first electrokinetically differentiated and largely separated within a single channel. The characteristic separation times spanned a few seconds to a few minutes time. This data supports the concept that complex bioparticles can be identified by their electrical properties in short periods of time and for low-abundance samples. This approach could transform current medical diagnostics by eliminating the need for time-consuming steps (culturing, genotyping, resistance panels, *etc*.) in the clinical pathology workflow.

This concept is supported by both interrogations. The single-strain experiments revealed a significant difference in *V*_A_ required for capture of each strain. Calculated values for *c* were 443 ± 59 V and 881 ± 38 V for the resistant and susceptible strains, respectively. These values for *c* correspond to *μ*_EK_/*μ*_DEP_ values of 4.6 ± 0.6 × 10^9^ V m^–2^ and 9.2 ± 0.4 × 10^9^ V m^–2^. Note the same fluorescent chemical label was used for both strains, eliminating this as a potential differentiator. This difference indicates that the two analytes’ ratios of *μ*_EK_/*μ*_DEP_ are sufficiently distinct for separation. Interestingly, the analytes could still prove differentiable if they shared the same value for *c*, but different accretion slopes for *V*_A_ > *c*. In this latter scenario, electrokinetic velocity of the two analytes would serve as the primary differentiating factor.

The dual-strain experiments demonstrate a proof-of-principle separation of the two strains within a single g-iDEP microchannel. These experiments revealed significantly different loci of capture for the two strains within V2 microchannels. Qualitatively, the observed order of capture within the V2 microchannels corresponded with inferences drawn from the single-strain data regarding relative electrokinetic and dielectrophoretic mobilities. Specifically, gentamicin-resistant *S. epidermidis* (red labeled) were captured at larger-pitch gates and gentamicin-susceptible bacteria (green labeled) were captured at smaller-pitch gates. Thus, the ratio *μ*_EK_/*μ*_DEP_ is expected to be larger for gentamicin-susceptible than for gentamicin-resistant *S. epidermidis*. This is a significantly different result than previous bacterial strain differentiations,^26,45^ since the strains were physically separated and concentrated as opposed to differentiated *via* dielectrophoretic forces.

The separation of *S. epidermidis* strains was not complete; there were overlapping zones with some admixture of the two strains. However, this does not reflect limitations to the technique, but in the current ‘first generation’ designs. These limitations and possibilities for their reduction are discussed below. Separate and chemically distinct dyes were used for the dual strain experiment, potentially allowing the labeling strategy to influence the separation. Both dyes were attached using the same linker, which reacts with exposed primary amines. Thus, no significant effects are expected from the linker system. The two fluorescent moieties (rhodamine and fluorescein) differ in pI and therefore may influence particle surface properties as well as the separation. However the difference between these two dyes as implemented in these experiments is expected to reduce rather than enhance separation. Since pI_fluorescein_ < pI_rhodamine_, any differential effects upon the particles’ electrokinetic mobilities would bring their respective mobility ratios into closer proximity. Dye reversal studies are planned, but the single strain data and this dual strain data already demonstrate unequivocal differentiation.

In these experiments, a distinct and statistically significant difference was observed between the behavior of gentamicin-resistant and gentamicin-susceptible *S. epidermidis*. The physical and structural differences associated with gentamicin resistance and susceptibility may be subtle, but they are sufficient to facilitate separation. The physical origins and effects stem from the structural and molecular elements of cells. In gram-positive cocci such as *S. epidermidis* the cell envelope primarily consists of two layers: an outer, thick peptidoglycan layer and an inner cell membrane (Fig. 2). Sandwiched between these two layers is a thin periplasmic space. Electromotive forces depend upon complex and subtle variables; bacteria and other cells are especially complex entities from an electrophysical point of view. They consist of multiple subdomains that all possess independent or semi-independent electric and dielectric properties.^53^ These subdomains are never spherical, lossless, or isotropic (as is often presumed for theoretical treatment of electrokinetic forces). Living cells, for instance, consist of multiple aqueous regions separated by semipermeable membranes. The lipid membrane itself is composed of polar molecules and contains highly peripatetic membrane-bound proteins. Internal structures such as the cytoskeleton and organelles are also polarizable, mobile or semi-mobile, and likely contribute to the overall multipolar character of the cell. These characteristics can vary between biological targets, even based on slight differences in genotype.

Changes in surface features such as the peptidoglycan layer, surface-expressed proteins, or teichoic acids are likely to influence electrophoretic mobility.^54^ Constituents of the cell wall (including proteins, lipids, and polysaccharides), the permeability of the cell wall, and internal cytoplasmic structures are all likely to affect dielectrophoretic mobility. One direct mechanism for physical cellular change could be overexpression of the AAC(6′)-APH(2′′) bifunctional enzyme. The isoelectric points of AAC(6′) and APH(2′′) have been shown to range from approximately 5 to 8.^55^ This differs greatly from the pI of *S. epidermidis*, which is 2.3.^56^ If these are expressed on the cell surface, there could be a direct electrophoretic effect since the pI of the bacteria would be significantly altered. It is noteworthy that osmotic shock studies with resistant *E. coli* bacteria indicate some gentamicin resistance-conferring enzymes may be more concentrated within the cell envelope, in particular the periplasmic space.^57^

Recognizing that subtle changes in a cell's envelope, inner structure, overall shape, or deformability can result in a unique net force on that cell, it is reasonable to expect genetically or phenotypically distinct strains to behave differently in response to electric fields. The complex dielectric characteristics of a biological cell and its interactions with the surrounding medium are approximated by an experimental or effective value for the Clausius–Mossotti factor (*f*_CM_), which is an important component of the dielectrophoretic force equation. The smallest theoretically resolvable difference for the *f*_CM_ is about one part in 10^5^ under the conditions of these experiments.^52^ If presumed to represent only changes in effective cell conductivity,^45^ this could translate to changes as small as a few μS m^–1^. Castellarnau *et al.* estimated that cell wall and membrane conductivities vary up to 70% for isogenic mutants of a single strain of *E. coli*.^58^ There are many examples in the literature where small changes in molecular structure of cells generate electrophysical differences, sometimes used for separations.^19–34^ Based their results, previous g-iDEP results with strains of *E. coli*, and theoretical resolution estimates, the observed differences in electromotive behavior can reasonably be attributed to mechanisms associated with gentamicin resistance in *S. epidermidis*.

When capture occurs, a large variability in signal was generally observed (Fig. 4). The data were obtained over several months, on many devices, and by different operators. While the assessed error appears to be large, it does not preclude establishing initiation of capture (*c*) and approximating a slope (*m*) of *N vs. V*_A_, the key elements of this study. Variations between experimental sessions in the following parameters may contribute to the spread: specific bacterial cells counts, staining efficiency, photobleaching, and slight pressure-driven or electroosmotic flow bias. The effects of these variables are compounded by the amount of material captured and measured at the ROI. Thus the standard deviation appears to increase proportionally with *V*_A_. One possibility is that natural biological variations contribute significantly to the total variance. Independent assessment of cell diameter, surface area, surface roughness, *etc*. would be required to begin to tease this out,^34^ as well as quantitation of dispersive forces within each gate area (a current topic of investigation—comparing generations of g-iDEP devices).

For dual-strain experiments, these sources of variability also hold (Fig. 5). These can be attributed largely to two phenomena: the increased resolution of the V2 channels compared to the V1 channels, and low capture efficiency at any given gate. The latter results from the dispersive effect of transverse electric field inhomogeneity, especially across the gate axis. This inhomogeneity is a consequence of the formation of extremely high gradient zones in the immediate vicinity of sharp geometric features. This lateral field inhomogeneity is being addressed with new device designs that will minimize these particular effects.

At high values of *V*_A_, detrimental and interfering effects are introduced by Joule heating and bubble formation. With alternative experimental or device design, capture could be achieved with lower applied potential; this would require either smaller gate pitch or a reduction in EK velocity.

The current device operates in an analytical mode, it simply separates the strains as a method of identification. However, it can also serve as a sample preparation module where the collected fractions are ported off the main channel with orthogonal side channels. These side channels can be held electrically silent during capture and then activated to draw the concentrated bolus to another section of the chip or off chip for further analysis (mass spectrometry, PCR, phenotyping, culturing, *etc*.).

With adequate resolution and dynamic range, it is reasonable to expect that a g-iDEP microchannel will generate unique loci for separation and concentration of multiple bioanalytes. Furthermore, these bioanalytes may range from dissimilar to similar, spanning a variety of clinically important targets. The present implementation of g-iDEP has already shown sufficient resolution for differentiating pathogenic and non-pathogenic strains of *E. coli*. The results presented here break new ground by differentiating and separating bacteria based upon their antibiotic susceptibility. While the physical forces at work are unlikely sufficient to observe simple mutations in the genetic code, it is plausible that any expressed gene product will alter the physicochemical parameters of the cell in a sufficient manner to effect separation. With the potential for extremely high resolution and large dynamic range, this strategy will create a new and extremely valuable tool for identifying and isolating pathogens. Additionally, this tool could be used as a powerful preparative step for other traditional modes of characterization. In these cases, g-iDEP would offer improved results obtained from traditional methods by first removing interfering components and concentrating the target.

## Conclusion

Using two types of sawtooth-patterned g-iDEP microchannels, this work demonstrates both differentiation and spatial resolution of gentamicin-resistant and gentamicin-susceptible *S. epidermidis*. Importantly, this is achieved using DC fields and easily achievable values of applied potential.

Previous work in this field has demonstrated differentiation of similar bioparticles, including pathogenic and non-pathogenic strains of *E. coli*. This research represents a refinement of the existing technique, and introduces the use of a higher-resolution g-iDEP sawtooth microchannel to effect the separation. These results bear significant implications for the future of clinical analytics and diagnostics. Additional modeling and refinements of g-iDEP microchannel geometry will improve the resolution and capabilities of this technique.

## Abbreviations

g-iDEP: Gradient insulator-based dielectrophoresis
iDEP: Insulator-based dielectrophoresis
DEP: Dielectrophoresis
EP: Electrophoresis
EOF: Electro-osmotic flow
*μ*_EK_: Electrokinetic mobility
*μ*_EP_: Electrophoretic mobility
*μ*_EOF_: Electroosmotic mobility
*μ*_DEP_: Dielectrophoretic mobility
*f*_CM_: Clausius–Mossotti factor
