# Correction: Biophysical separation of *Staphylococcus epidermidis* strains based on antibiotic resistance

**DOI:** 10.1039/c5an90100f

**Published:** 2015-12-02

**Authors:** Paul V. Jones, Shannon Huey Hilton, Paige E. Davis, Ryan Yanashima, Ryan McLemore, Alex McLaren, Mark A. Hayes

**Affiliations:** Arizona State University, Department of Chemistry and Biochemistry Tempe AZ 85287 USA mhayes@asu.edu +1 (480) 965-2747 +1 (480) 965-2566

## Abstract

Correction for ‘Biophysical separation of *Staphylococcus epidermidis* strains based on antibiotic resistance’ by Paul V. Jones *et al.*, *Analyst*, 2015, **140**, 5152–5161.

The authors regret the omission of one of the authors, Ryan Yanashima, from the original manuscript and that incomplete details were shown for Shannon Huey Hilton and Paige E. Davis. The corrected list of authors and affiliations for this paper is as shown above.

The Royal Society of Chemistry apologises for these errors and any consequent inconvenience to authors and readers.

## Supplementary Material

